# Do CO_2_ emissions, per capita GDP, public and private health expenditures matter for the health of older adults: empirical evidence from the E7 countries

**DOI:** 10.3389/fpubh.2025.1694795

**Published:** 2025-10-15

**Authors:** Muhammet Ali Köroğlu, Gamze Sart, Yilmaz Bayar, Marina Danilina

**Affiliations:** ^1^Department of Social Work, Faculty of Economics and Administrative Sciences, Usak University, Usak, Türkiye; ^2^Department of Educational Sciences, Hasan Ali Yucel Faculty of Education, Istanbul University-Cerrahpaşa, Istanbul, Türkiye; ^3^Department of Public Finance, Bandirma Onyedi Eylül University, Balikesir, Türkiye; ^4^Department of Economics, Plekhanov Russian University of Economics (PRUE), Moscow, Russia; ^5^Department of Economics, Financial University under the Government of the Russian Federation, Moscow, Russia

**Keywords:** CO_2_ emissions, per capita GDP, older adults’ health, panel econometrics, public health expenditures, private health expenditures

## Abstract

**Background:**

Improvements in life expectancy, along with decreases in fertility rates, have caused population aging in many countries. However, the environmental, economic, and social determinants of older adults’ health have not been sufficiently researched.

**Methods:**

The objective of this research is to explore the effects of CO_2_ emissions, per capita GDP, and public and private health expenditures on the health of older adults in E7 countries during the period 2000–2021, using causality and cointegration methods.

**Results:**

The findings of the JKS causality test indicate a reciprocal interaction among life expectancy at age 60 (LE60), healthy life expectancy at age 60 (HALE60), CO_2_ emissions, per capita GDP, and public and private health expenditures. Furthermore, the findings of the long-term analysis reveal that CO_2_ emissions negatively impact LE60 and HALE60, whereas per capita GDP and public and private health expenditures positively influence LE60 and HALE60.

**Conclusion:**

The findings of this study highlight that the stringency of environmental policies, development of renewable energy technologies, income equality, and efficiency of health and social security systems are crucial for improving LE60 and HALE60.

## 1 Introduction and background

In recent years, human longevity has increased globally as a result of advances in medicine, science, and economic and social development. In this regard, life expectancy at birth (LEB) globally reached 73.3 years in 2024 from 64.9 years in 1995 (1). However, fertility rates have declined in many countries, mainly because of economic insecurity, higher education, urbanization, family postponement, and an increase in women’s employment (2). Consequently, the phenomenon of population aging has begun to be witnessed in many countries, and the number of people aged 60 years and older is forecasted to increase from 1.1 billion in 2023 to 1.4 billion by 2030 (1). In addition, many countries have increased their retirement age in parallel with an increase in life expectancy.

Increases in life expectancy are significant for economic growth through human and physical capital investment channels. On the one hand, longer life expectancy positively affects savings and, in turn, stimulates economic growth by increasing physical capital investment, as suggested by neoclassical and endogenous growth models (3). Moreover, improvements in life expectancy imply higher returns on human capital, which can foster economic growth by encouraging further investments in education and skill development (3). However, empirical studies on the growth effects of life expectancy remain inconclusive (4–8).

In light of the above considerations, promoting the health of older adults is crucial for maintaining active participation in the family and community. In this regard, air pollution is one of the major environmental risks to health, and nine out of ten people live in a low air quality environment. Approximately seven million people worldwide die annually from air pollution (9). Furthermore, studies on the impact of air pollution on cardiovascular diseases (CVDs) have revealed a strong relationship between short- and long-term air pollution exposure and CVD morbidity and mortality, blood pressure, ischemic heart disease, and stroke (10). Therefore, CO₂ emissions, primarily produced by fossil fuel combustion, industrial production, transportation, and electricity generation, are among the dominant drivers of air pollution, global warming, and climate change. Climate change is another fundamental threat to human health. More frequent and intensifying weather events, such as floods, storms, extreme heat, wildfires, and droughts, resulting from global warming and climate change, may increase the risk of noncommunicable diseases and deaths, the emergence and spread of infectious diseases, and health emergencies (11). As a result, climate change is predicted to lead 250,000 additional deaths per year through heat stress from undernutrition, malaria, and diarrhea between 2030 and 2050 (11). In summary, CO₂ emissions may negatively affect human health through multiple channels, including air pollution, increased temperatures, and climate change (12). Conversely, an increase in life expectancy can contribute to higher CO₂ emissions by prolonging individuals’ economic and social activities. Thus, a feedback relationship between life expectancy and CO₂ emissions is theoretically plausible.

Relatively higher income levels enable individuals to access goods and services, such as nutritious food, improved sanitation, safe water, and higher-quality medical care, all of which support longer and healthier lives (12). Preston (13) examined the relationship between life expectancy at birth (LEB) and per capita GDP during the 1900s, 1930s, and 1960s and suggested that individuals in countries with relatively higher per capita GDP generally had higher life expectancies. However, the strength of this relationship diminishes over time. In other words, countries with low per capita GDP experienced greater increases in LEB as their income levels increased, whereas countries with high per capita GDP recorded relatively smaller gains. The outward shift of the Preston curve was attributed to exogenous factors such as improvements in nutrition and literacy (13). Consequently, a positive influence of per capita GDP on LEB is theoretically expected. Conversely, increases in the LEB are also expected to affect per capita GDP through physical and human capital channels.

Finally, health expenditure can positively affect life expectancy by providing higher-quality healthcare services through improved medical facilities and better physicians (14). Moreover, spending on preventive measures, curative care, nutrition, and vaccination can enhance life expectancy (15). Conversely, increases in life expectancy may lead to higher health expenditures as older individuals typically require more intensive healthcare (16).

The main objective of this study is to analyze the effects of CO₂ emissions, per capita GDP, and public and private health expenditures on the health of older adults, measured by life expectancy at age 60 (LE60) and healthy life expectancy at age 60 (HALE60) in the E7 countries—Brazil, China, India, Indonesia, Mexico, Russia, and Türkiye—which represent the strongest economic performers among emerging economies. As shown in Table 1, China, India, Indonesia, and Türkiye experienced substantial increases in both per capita CO₂ emissions and per capita GDP between 2000 and 2021. However, the per capita health expenditures of E7 countries generally lagged behind those of developed countries during this period. In particular, per capita health expenditures in India, Indonesia, and Türkiye will remain very low, amounting to US$76, US$159, and US$431, respectively, in 2021. Over the study period, only China achieved significant progress in LE60 and HALE60, largely because of notable increases in per capita GDP and health expenditure.

**Table 1:** LE60, HALE60, CO_2_, PCGDP as per capita GDP, and PCHEX as per capita current health expenditures in E7 countries (2000, 2021).

Countries	Years	LE60	HALE60	CO_2_	PCGDP	PCHEX
Brazil	2000	19.78	14.68	1.8	6817.784	314
	2021	19.41	14.35	2.22	8799.229	768
China	2000	17.96	13.98	2.63	2237.443	43
	2021	21.21	16.19	8.06	11469.57	670
India	2000	17.51	12.7	0.89	756.7041	18
	2021	15.62	11.53	1.75	1965.309	76
Indonesia	2000	17.35	13.24	1.3	1828.103	15
	2021	15.4	11.86	2.13	3850.689	159
Mexico	2000	21.44	15.93	3.84	9581.97	319
	2021	18.51	13.87	3.19	9728.057	606
Russian Federation	2000	16.39	12.28	10.66	5323.667	95
	2021	16.96	12.69	11.94	10231.32	882
Türkiye	2000	20.32	15.42	3.31	6325.096	193
	2021	19.32	14.6	5.29	13449.93	431

Source: WHO (53), Climate Watch (54), World Bank (56), WHO (55).

In the empirical literature, most studies have examined the relationship between CO₂ emissions, per capita GDP, health expenditures, and life expectancy at birth (LEB), with the exception of Aytemiz et al. (17) and Magazzino et al. (18). Therefore, this study is among the first to investigate the effects of CO₂ emissions, per capita GDP, and health expenditures on the health of older adults. Moreover, while scholars have typically focused on the impact of CO₂ emissions, per capita GDP, and health expenditures on LEB, the reverse effects of life expectancy on these variables have not been adequately explored. Hence, the second contribution of this study is to conduct a two-way analysis of CO₂ emissions, per capita GDP, public and private health expenditures, and LE60/HALE60.

The next section presents a summary of the empirical literature on LE60, HALE60, CO₂ emissions, per capita GDP, and health expenditures. Section 3 outlines the study’s dataset and methodology. Section 4 reports the econometric tests and discusses the results of the study. Finally, Section 5 concludes the study.

## 2 Literature overview

The proportion of older people in the world’s population has been increasing; however, the determinants of older adults’ health have not been adequately researched. The relatively few empirical studies available suggest that income level, financial security, physical and social environments, access to health services, physical activity, lifelong learning, and personal factors are significant determinants of older adults’ health (19–21). Building on this limited literature, this study examines the relationship between older adults’ health, CO₂ emissions, per capita GDP, and health expenditures.

In the associated literature, academicians have usually explored the connection between CO_2_ emissions and LEB, as shown in Table 2. In this regard, the vast majority of studies, including those in references (14, 22–34) identified a negative connection between CO_2_ emissions and LEB, whereas the studies in references (35–37) uncovered a positive connection between CO_2_ emissions and LEB. On the other hand, Das and Debanth (38) and Azam and Adeleye (39) disclosed mixed results, and Redzwan and Ramli (40) and Hasnawati et al. (41) found an insignificant connection between the two variables. Furthermore, Rahman et al. (23), Javanshirova (26), and Rjoub et al. (35) determined a causal connection from CO_2_ emissions to LEB, and Khan et al. (34) disclosed a bilateral causal connection between LEB and carbon dioxide emissions, while Hasnawati et al. (41) unveiled an insignificant interplay between the two variables.

**Table 2:** Literature on the connection between CO_2_ emissions and life expectancy.

Study	Countries; period	Methods	Influence of CO_2_ emissions on health indicators; causality; health indicator
Anwar et al. (14)	OECD member; 1996–2020	Regression	Negative; LEB
Chen et al. (22)	20 countries; 2004–2016	Regression	Negative (developing and developed countries; LEB)
Rahman et al. (23)	Most polluted countries; 2000–2017	Regression and causality methods	Negative, unidirectional causal connection from CO_2_ emissions to LEB
Nwani and Imhanzenobe (24)	Nigeria	Structural equation modelling	Negative; LEB
Radmehr and Adebayo (25)	Mediterranean countries; 2000–2018	Regression	Negative; LEB
Javanshirova (26)	Azerbaijan; 1974–2022	ARDL	Negative; unilateral causal connection from CO_2_ emissions to LEB
Khan et al. (27)	India; 2000–2020	ARDL	Negative; LEB
Saidmamatov et al. (28)	Aral Basin states; 2002–2020	DOLS, FMOLS, and Driscoll–Kraay estimators	Negative; LEB
Țarcă et al. (29)	13 Eastern European States; 2000–2020	Regression	Negative; LEB
Szymańska (30)	27 EU states; 1995–2019	Regression	Negative; LEB
Altaee et al. (31)	GCC states; 1990–2020	Regression	Negative; LEB
Zhang et al. (32)	China; 2000–2023	ARDL	Negative; LEB
Wahyudi and Leny (33)	Indonesia; 2010–2021	Sobel test	Negative; LEB
Khan et al. (34)	Developing economies; 1981–2020	ARDL	Negative; bidirectional causal link between LEB and CO_2_ emissions
Rjoub et al. (35)	Türkiye; 1960–2018	Cointegration and causality methods	Positive; unidirectional causal link from CO_2_ emissions to LEB
Mahalik et al. (36)	68 emerging and developing economies; 1990–2017	Regression	Positive (developing economies) and negative (emerging economies); LEB
Osei-Kusi et al. (65)	82 countries; 1990–2020	Regression	Positive; LEB
Yang and Ying (37)	India; 1990–2020	Regression	Positive; LEB
Das and Debanth (38)	India; 1991–2018	ARDL	Positive until a certain threshold level of CO_2_ emissions, but then negative; LEB
Azam and Adeleye (39)	36 countries; 2005–2010	Regression	Differs depending on the income levels of the countries; LEB
Redzwan and Ramli (40)	Malaysia; 1997–2021	ARDL	Insignificant; LEB
Hasnawati et al. (41)	Indonesia; 1950–2020	Causality	Insignificant causality; LEB

Source: Authors’ own elaboration.

The following hypotheses were identified based on theoretical views and empirical literature evidence:

*Hypothesis 1*. CO_2_ emissions negatively impact LE60 and HALE60 in the long term.

*Hypothesis 2*. There exists a feedback interplay between CO_2_ emissions, LE60, and HALE60.

Income level is one of the dominant determinants of public health, and in turn, nearly all studies introduced in Table 3, such as Anwar et al. (14), Aytemiz et al. (17), Magazzino et al. (18), Chen et al. (22), Radmehr and Adebayo (25), Țarcă et al. (29), Miladinov (42), Şenol et al. (43), Shi et al. (44), Morina et al. (45), and Karunarathne et al. (46), revealed a positive link between per capita GDP and LEB. Meanwhile, Magazzino et al. (18) found unidirectional causality from LEB, LE40, and LE60 to per capita GDP, and Alwago (47) found an insignificant causal link between LEB and per capita GDP.

**Table 3:** Literature on the connection between income level and life expectancy.

Study	Countries; period	Methods	Influence of per capita GDP on health indicators; causality; health indicator
Aytemiz et al. (17)	OECD states; 2005–2021	Cointegration	Positive; LEB, HALEB, LE60, and HALE60
Magazzino et al. (18)	OECD members; 1998–2018	Causality and machine learning	Positive; unidirectional causality from LEB, LE40, and LE60 to per capita GDP
Chen et al. (22)	20 countries; 2004–2016	Regression	Positive (developed countries) and negative (developing countries); LEB
Radmehr and Adebayo (25)	Mediterranean countries; 2000–2018	Regression	Positive; LEB
Țarcă et al. (29)	13 Eastern European States; 2000–2020	Regression	Positive; LEB
Miladinov (42)	EU accession candidates; 1990–2017	Regression	Positive; LEB
Şenol et al. (43)	49 countries; 2000–2019	Regression	Positive; LEB
Shi et al. (44)	Finland; 1996–2017	Regression	Positive; life tables for ages 25+
Morina et al. (45)	OECD; 2005–2018	Regression	Positive; LEB
Karunarathne et al. (46)	20 low-income countries; 2000–2021	Regression	Positive; LEB
Alwago (47)	Kenya; 2000–2020	Causality test	Insignificant causality between GDP growth and LEB

Source: Authors’ own elaboration.

The following hypotheses are identified based on theoretical views and empirical literature evidence:

*Hypothesis 3*. Per capita GDP positively impacts LE60 and HALE60 in the long term.

*Hypothesis 4*. There exists a feedback interplay between per capita GDP, LE60, and HALE60.

Health expenditure is also one of the crucial factors behind public health, together with per capita GDP. The literature summary in Table 4 indicated that nearly all studies, including Anwar et al. (14), Bayar et al. (16), Aytemiz et al. (17), Radmehr and Adebayo (25), Morina et al. (45), Rezapour et al. (48), Bunyaminu et al. (49), Sarıyıldız (50), and Alimi et al. (51), found a positive connection between diverse indicators of health expenditures and LEB, while Saraç and Torun (52) disclosed an insignificant link between LEB and public and private health expenditures. Bayar et al. (16) also discovered a unidirectional LEB for health expenditure.

**Table 4:** Literature on the connection between health expenditure and life expectancy.

Study	Countries; period	Methods	Influence of health expenditures on health indicators (proxy of health expenditures); causality; health indicator
Bayar et al. (16)	EU members; 2000–2018	Causality test	Unidirectional LEB to health expenditures
Aytemiz et al. (17)	OECD members; 2005–2021	Cointegration test	Positive (current health expenditures); LEB, HALEB, LE60, and HALEB60
Radmehr and Adebayo (25)	Mediterranean countries; 2000–2018	Regression	Positive; LEB
Morina et al. (45)	OECD; 2005–2018	Regression	Positive (health expenditures); LEB
Rezapour et al. (46)	105 countries; 2000–2015	Regression	Positive (public health expenditure); LEB
Bunyaminu et al. (49)	African states; 2000–2018	Regression	Positive (health expenditures); LEB
Sarıyıldız (50)	Türkiye; 2001–2021	Regression	Positive (per capita healthcare expenditures); LEB
Alimi et al. (51)	Nigeria; 1984–2020	ARDL	Negative (short-term) and positive (long-term) (government health expenditures); LEB
Saraç and Torun (52)	Türkiye; 2002–2019	Regression	Insignificant (both public and private health expenditures); LEB

Source: Authors’ own elaboration.

The following hypotheses are identified based on theoretical views and empirical literature evidence:

*Hypothesis 5*. Public health expenditure positively impacts LE60 and HALE60 in the long term.

*Hypothesis 6*. Private health expenditure positively impacts LE60 and HALE60 in the long term.

*Hypothesis 7*. There exists a feedback interplay between public health expenditure, LE60, and HALE60.

*Hypothesis 8.* There exists a feedback interplay between private health expenditure, LE60, and HALE60.

## 3 Data and methods

This study delves into the influence of CO_2_ emissions, per capita GDP, and public and private health expenditures on older adults’ health in E7 countries by utilizing causality and cointegration tests. In the analysis, the health state of older adults is represented by LE60 and HALE60, which are sourced from the WHO (53). On the other hand, environmental impairment is proxied by CO emissions per capita (tCO_2_ equivalent per capita) (CARBON), and these data are sourced from Climate Watch (54). Health expenditures are substituted by domestic general government (% of current health expenditures) (GGHEX) and domestic private health expenditures (% of current health expenditures) (PHEX), which indicate the share of current health expenditures funded by public and domestic private sources, respectively, as a percentage of current health expenditures. Domestic private health expenditures consist of prepaid (such as voluntary and compulsory prepayments to private insurance or paid directly out-of-pocket by households) and demonstrate the share of domestic private sources, such as domestic revenues of households, corporations, and non-profit organizations, in funding healthcare relative to government or external sources. Both indicators are sourced from the WHO (55). Finally, income level is substituted via per capita GDP (2015 US$) (PCGDP), and these data are sourced from the World Bank (56).

The dataset consists of seven emerging markets, and the time dimension of the dataset covers the years between 2000 and 2021 because indicators of LE60 and HALE60 are present between 2000 and 2021. In econometric applications, tests of cross-section dependency (CD), unit root, heterogeneity, causality, and AMG estimation are carried out in favor of Stata 17.0, and the cointegration test is performed using Gauss 12.0.

The main aim of this study is to investigate the interplay between the indicators of LE60 and HALE60, CO_2_ emissions, per capita GDP, and public and private health expenditures. Consequently, the two models in Equations 1, 2 are used for the empirical analyses:


(1) $$\begin{array}{l} \mathrm{LE}60_{\mathrm{it}}=\alpha_{0}+\beta_{1}\text{CARBO}N_{\mathrm{it}}+\beta_{2}\text{PCGD}P_{\mathrm{it}}+\beta_{3}\text{GGHE}X_{\mathrm{it}}\\ +\beta_{3}\mathrm{PHE}X_{\mathrm{it}}+u_{\mathrm{it}}\;\;(\text{Model}-1) \end{array}$$

(2) $$\begin{array}{l} \text{HALE}60_{\mathrm{it}}=\alpha_{0}+\beta_{1}\text{CARBO}N_{\mathrm{it}}+\beta_{2}\text{PCGD}P_{\mathrm{it}}+\beta_{3}\text{GGHE}X_{\mathrm{it}}\\ +\beta_{3}\mathrm{PHE}X_{\mathrm{it}}+u_{\mathrm{it}}\;\;(\text{Model}-2) \end{array}$$

The mean values of LE60 and HALE60, presented in Table 5, are 19.378 and 14.559 years, respectively. Additionally, the average values of CARBON, PCGDP, GGHEX, and PHEX are 4.313 metric tons per capita, US$6562.619, 47.903, and 51.682%, respectively. However, per capita GDP and public and private health expenditures show very high variability among the E7 states, whereas LE60, HALE60, and CARBON display moderate variation.

**Table 5:** Descriptive statistics of LE60, HALE60, CARBON, PCGDP, GGHEX, and PHEX (2000–2021).

Series	Averaged values	Std. dev.	Minimum	Maximum
LE60	19.378	1.782	15.4	22.28
HALE60	14.559	1.334	11.53	16.58
CARBON	4.313	3.285	0.89	11.94
PCGDP	6562.619	3437.921	756.704	13449.93
GGHEX	47.903	16.480	17.982	80.499
PHEX	51.682	16.074	19.501	79.783

Source: Authors’ own elaboration.

Westerlund and Edgerton’s (57) cointegration test considers CD, heterogeneity, autocorrelation, and structural breaks during the dataset period. The cointegration coefficients of the panel and each E7 country are forecasted using the AMG estimator proposed by Eberhart and Bond (58). Traditional estimators do not consider heterogeneity and CD; therefore, their results can be spurious and biased (59). However, the AMG estimator considers CD, heterogeneity, and endogeneity differently from first-generation estimators (58). Additionally, the AMG estimator can make an estimation at both the panel and country levels. The AMG estimator utilizes Equation 3 and considers CD by attaching a common dynamic effect elaborated from the dummy coefficients of the regression in the first differences to group-specific regressions (58).


(3) $$y_{\mathrm{it}}=\beta_{i}^{\prime }x_{\mathrm{it}}+u_{\mathrm{it}}\;u_{\mathrm{it}}=\alpha_{i}+\lambda_{i}^{\prime }f_{t}+\varepsilon_{\mathrm{it}}$$

(4) $$x_{\mathrm{mit}}=\pi_{\mathrm{mi}}+\delta_{\mathrm{mi}}^{\prime }g_{\mathrm{mt}}+\rho_{1\mathrm{mi}}f_{1\mathrm{mt}}+\ldots +\rho_{\mathrm{nmi}}f_{\mathrm{nmt}}+v_{\mathrm{mit}}$$

where the observable covariates ($x_{\mathrm{it}}$) and unobservable covariates ($u_{\mathrm{it}}$) are specified as a combination of group effects ($\alpha_{i}$), common factors ($f_{t}$), and group factor loadings ($\lambda_{i}^{\prime }$).

The causal relationships among LE60, HALE60, CARBON, PCGDP, GGHEX, and PHEX are examined using the JKS (60) causality method. The JKS causality test has several advantages over the Granger causality test. The test considers heterogeneity and CD, unlike the Granger causality test, and also takes advantage of the half-panel jackknife (HPJ) approach proposed by Dhaene and Jochmans (61) to decrease Nickell bias (60). Furthermore, the JKS causality test displays robustness with unbalanced panels and smaller sample sizes compared to some alternatives (62).

## 4 Results

In the first stage of the econometric analyses, the subsistence of heterogeneity and CD are analyzed in favor of the tests of delta and LM (Lagrange multiplier), respectively. In this sense, CD tests of LM_adj._, LM CD, and LM tests are conducted, and their test statistics are presented in Table 6. The H_0_ hypothesis of CD independence is rejected by considering their probability values lower than 0.01. Similarly, homogeneity tests of delta are applied, and their test statistics are presented in Table 6. The H_0_ hypothesis of homogeneity is rejected by considering their probability values lower than 0.01. The consequences of the delta and CD tests testify to the subsistence of heterogeneity and CD among LE60/HALE60, CARBON, PCGDP, and PCHEX in Model-1 and Model-2 and suggest that we utilize econometric tests sensitive to heterogeneity and CD.

**Table 6:** Outcomes of the CD and slope homogeneity tests.

CD tests	Test statistic		Homogeneity tests	Test statistic	
	Model-1	Model-2		Model-1	Model-2
LM	51.53***	53.51***	Delta	6.084***	6.124***
LM_adj._	8.633***	9.287***	Bias-Adj. Delta	7.134***	7.181***
LM CD	4.811***	5.034***			

Source: Authors’ own elaboration. *** Significant at 1%.

In addition, the integration of levels of the series in Models 1 and 2 should be identified before the selection of cointegration and causality methods. Therefore, the CIPS unit root test of Pesaran (63) is performed to identify the integration levels of LE60, HALE60, CARBON, PCGDP, GGHEX, and PHEX, and its findings are reported in Table 7. The test statistics of the CIPS test disclose that LE60, HALE60, CARBON, PCGDP, GGHEX, and PHEX include a unit root at level values, but these series become stationary when the test is run with the first differences of LE60, HALE60, CARBON, PCGDP, GGHEX, and PHEX.

**Table 7:** CIPS test’s findings.

Series	Level		First differences	
	Constant	Constant + Trend	Constant	Constant + Trend
LE60	−0.524	1.167	−4.622***	−2.753***
HALE60	0.212	1.983	−4.663***	−5.031***
CARBON	1.848	2.767	−4.616***	−4.067***
PCGDP	−0.514	3.055	−3.203***	−3.563***
GGHEX	1.408	1.192	−4.645***	−6.0781***
PHEX	1.594	1.884	−5.348***	−4.254***

Source: Authors’ own elaboration. ***Significant at 1%.

The long-run interplay between LE60/HALE60, CARBON, PCGDP, GGHEX, and PHEX is questioned through the cointegration test of (57), and the test statistics along with the structural breaks are reported in Table 8. The models with level and regime shifts indicate the rejection of the H_0_ hypothesis of insignificant cointegration among the series in Model-1 and Model-2, while the model with no shift shows the acceptance of the null hypothesis of insignificant cointegration. Therefore, the utilization of the cointegration test with structural breaks is evaluated to increase the robustness of our outcomes. Additionally, structural breaks in 2004, 2005, 2008, 2010, 2017, and 2018 are endogenously specified in the model. These structural breaks indicate that the Brazilian currency crisis of 2002, the 2008 global financial crisis, the Eurozone sovereign debt crisis of 2010, the 2014 Brazilian economic crisis, the Russian financial crisis (2014–2016), the 2015–2016 Chinese stock market turbulence, and Türkiye’s ongoing major economic problems as of 2016 significantly impacted life expectancy and economic indicators in the country.

**Table 8:** Outcomes of the cointegration tests.

Test statistics	No shift (*p* values)	Level shift (*p* values)	Regime shift (*p* values)
Model-1			
Z_ϕ_ (N)	1.172 (0.879)	−2.385 (0.009)	−2.416 (0.008)
Z_τ_ (N)	2.162 (0.985)	−1.745 (0.040)	−2.082 (0.019)
Countries		Structural breaks	Structural breaks
Brazil		2004	2017
China		2005	2005
India		2008	2018
Indonesia		2004	2004
Mexico		2005	2017
Russian Federation		2010	2018
Türkiye		2017	2017
Model-2			
Z_ϕ_ (N)	1.103 (0.865)	−2.406 (0.008)	−1.768 (0.039)
Z_τ_ (N)	1.987 (0.977)	−2.571 (0.048)	−3.415 (0.000)
Countries		Structural breaks	Structural breaks
Brazil		2004	2017
China		2005	2005
India		2008	2018
Indonesia		2004	2004
Mexico		2005	2017
Russian Federation		2010	2018
Türkiye		2017	2017

Source: Authors’ own elaboration.

The cointegration coefficients of the explanatory variables in Model-1 and Model-2 are forecast in favor of the AMG estimator and are reported in Table 9. In addition, two models are forecasted by the Common Correlated Effects Mean Group (CCE-MG) estimator of Pesaran (64) to check the consistency and reliability of the estimations by the AMG estimator, and their results are introduced in Table 10.

**Table 9:** Cointegration coefficients using the AMG estimator.

Countries	CARBON		PCGDP		GGHEX		PHEX	
	LE60	HALE60	LE60	HALE60	LE60	HALE60	LE60	HALE60
Brazil	−0.043**	−0.035*	0.225***	0.217***	0.860*	0.784*	1.503**	1.349**
China	−0.024***	−0.019**	0.139***	0.118***	0.447**	0.437**	0.438**	0.443**
India	−0.171***	−0.166***	0.147***	0.163***	0.235**	0.174*	0.631**	0.487**
Indonesia	−0.065*	−0.050*	0.049**	0.0382*	0.037	0.026	0.092	0.067
Mexico	- 0.159***	−0.151**	0.274***	0.256***	0.358	0.332	0.461	0.436
Russian Federation	−0.392***	−0.375**	0.357***	0.348***	1.192**	1.222**	0.618**	0.639**
Türkiye	0.092	0.061	0.077**	0.081***	1.675***	1.515***	0.651***	0.593***
Panel	−0.063***	−0.058***	0.159***	0.151***	0.265	0.217	0.356	0.302

Source: Authors’ own elaboration. ***, **, and * are significant at 1, 5, and 10%, respectively.

**Table 10:** Cointegration coefficients obtained using the CCEMG estimator.

Countries	CARBON		PCGDP		GGHEX		PHEX	
	LE60	HALE60	LE60	HALE60	LE60	HALE60	LE60	HALE60
Brazil	−0.035**	−0.026*	0.129**	0.140***	0.724**	0.682**	1.039**	0.974**
China	−0.030**	−0.029**	0.072**	0.074**	0.113**	0.095**	0.155**	0.152**
India	−0.193***	−0.176**	0.077**	0.088**	0.213**	0.164*	0.548**	0.432*
Indonesia	−0.050**	−0.044**	0.40***	0.335***	0.037	0.037	0.089	0.095*
Mexico	0.048	−0.038*	0.709***	0.679***	0.941	0.909	1.430***	1.393**
Russian Federation	−0.075**	−0.080	0.202***	0.190***	0.234***	0.169***	0.051**	0.014***
Türkiye	−0.115	−0.099	0.093**	0.103**	0.810**	0.722**	0.267**	0.242**
Panel	−0.057**	−0.051*	0.136**	0.125**	0.407***	0.370**	0.467**	0.428**

Source: Authors’ own elaboration. ***, **, and * are significant at 1, 5 and 10%, respectively.

The results of the AMG estimation reveal that CO_2_ emissions negatively impact LE60 and HALE60 at the panel level and in all E7 countries except Türkiye, while the findings of the CCE-MG estimation also largely support these findings, except in Mexico. CO_2_ emissions have the largest negative effect on LE60 and HALE60 in Russia and India, whereas the negative effect of CO_2_ emissions on LE60 and HALE60 is relatively much lower in China, Brazil, and Indonesia.

In contrast, per capita GDP positively influences both LE60 and HALE60 at the panel level and in all E7 countries. The findings of the CCE-MG estimation also substantially confirm these results. The per capita GDP has the largest health impact in the Russian Federation, Mexico, Brazil, India, and China, whereas the health impact of per capita GDP is relatively lower in Türkiye and Indonesia.

Finally, both general government and private health expenditures positively impact LE60 and HALE60 in Brazil, China, India, the Russian Federation, and Türkiye, but the relation between indicators of health expenditures and LE60 and HALE60 is insignificant at the panel level in Indonesia and Mexico. Nevertheless, the findings of the CCE-MG estimation indicate that the panel-level positive connection between indicators of health expenditures and LE60 and HALE60 is significantly positive. Furthermore, the positive effect of public health expenditures on LE60 and HALE60 is the largest in Türkiye and the Russian Federation and the lowest in India and China.

**Figure 1:**
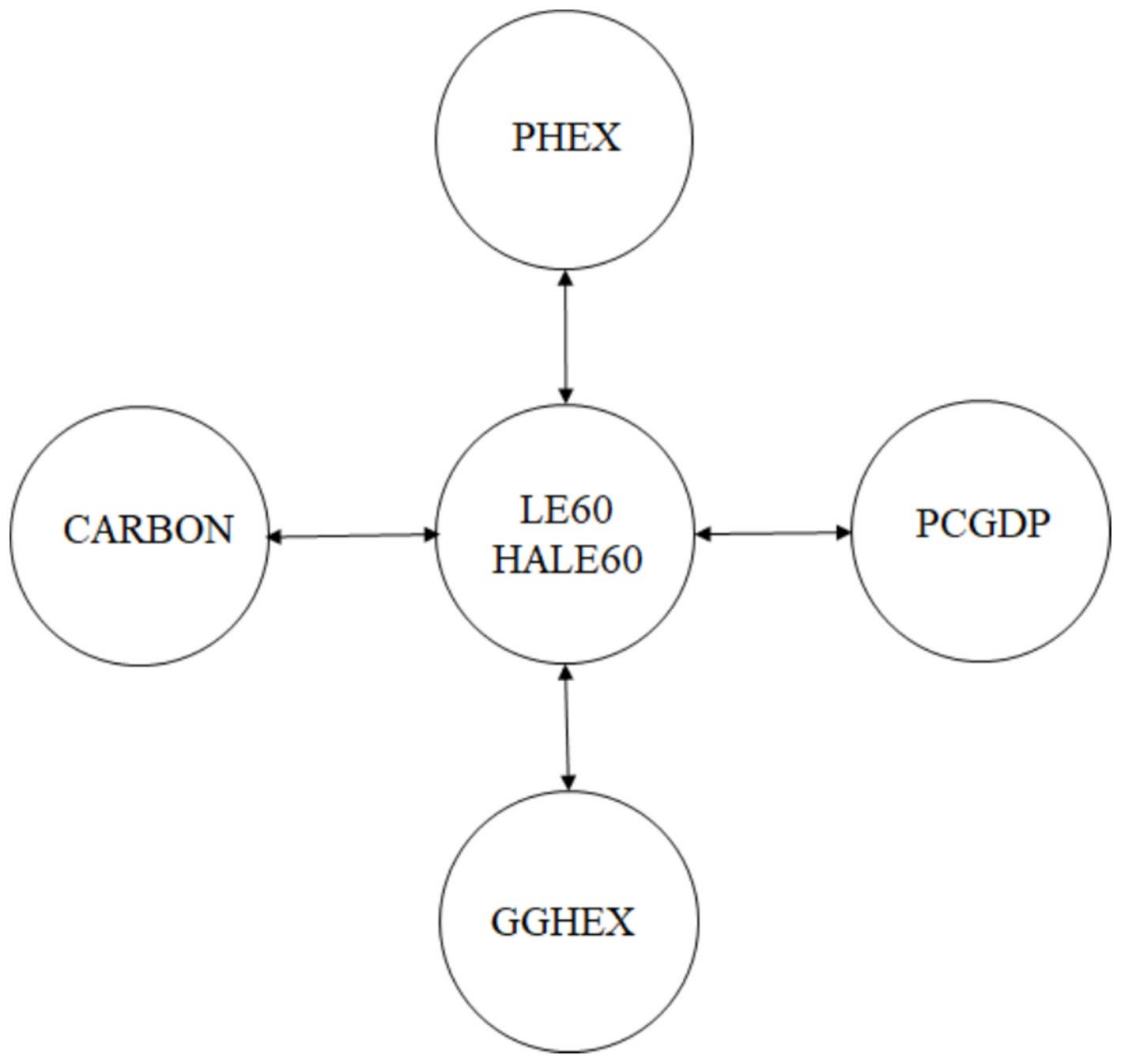
Causality test results. Source: Authors’ own elaboration.

The causal connection among CO_2_ emissions, per capita GDP, and indicators of health expenditures, LE60, and HALE60 is analyzed in favor of the JKS causality test, and the consequences of the test are shown in Table 11, Figure 1. The findings reveal a bidirectional causal link between CO_2_ emissions, per capita GDP, public and private expenditures, and LE60/HALE60.

**Table 11:** Findings of the JKS test.

Hypothesis	HPJ statistic
CARBON ⇏ LE60	38.2686***
LE60 ⇏ CARBON	8.8083***
CARBON ⇏ HALE60	12.0754***
HALE60 ⇏ CARBON	6.3773**
PCGDP ⇏ LE60	8.2072**
LE60 ⇏ PCGDP	24.7400***
PCGDP ⇏ HALE60	8.6865***
HALE60 ⇏ PCGDP	30.8757***
GGHEX ⇏ LE60	16.3261***
LE60 ⇏ GGHEX	64.5412***
GGHEX ⇏ HALE60	17.6435***
HALE60 ⇏ GGHEX	102.6688***
PHEX ⇏ LE60	15.6508***
LE60 ⇏ PHEX	55.4289***
PHEX ⇏ HALE60	16.6324***
HALE60 ⇏ PHEX	89.8210***

Source: Authors’ own elaboration. *** and ** are significant at 1 and 5%, respectively.

## 5 Discussion

On the one hand, CO_2_ emissions are anticipated to negatively influence the health of older adults through noncommunicable diseases, the emergence and spread of infectious diseases, and health emergencies mainly coming from air pollution, global warming, and climate change. However, improvements in life expectancy mean that people engage in CO_2_ emission-increasing activities for a longer period; hence, improvements in life expectancy may enhance CO_2_ emissions. However, the academicians have usually focused on the effect of CO_2_ emissions on LEB, and a negative connection between CO_2_ emissions and LEB has been verified by results of Anwar et al. (14), Chen et al. (22), Rahman et al. (23), Nwani and Imhanzenobe (24), Radmehr and Adebayo (25), Javanshirova (26), Khan et al. (27), Saidmamatov et al. (29), Țarcă et al. (29), Szymańska (30), Altaee et al. (31), Zhang et al. (32), Wahyudi and Leny (33), and Khan et al. (34). But Rjoub et al. (35), Mahalik et al. (36), Osei-Kusi et al. (65), and Yang and Ying (37) uncovered a positive connection between CO_2_ emissions and LEB. Our study investigates the connection between CO_2_ emissions and LE60 and HALE60 differently from the literature and discovered a negative effect of CO_2_ emissions on LE60 and HALE60 in all E7 countries except Türkiye, which is consistent with related theoretical views. The E7 countries experienced significant economic expansion along with lax environmental policies during the study period. Hence, the average values of the environmental policy stringency indices of these countries between 2016 and 2020 [The index ranges between 0 (not stringent) to 6 (highest degree of stringency)] are Mexico (0.167), Brazil (0.889), Russian Federation (1.168), Indonesia (1.633), India (2.656), Türkiye (2.794), and China (2.911) (66). Furthermore, the share of renewable energy use in the total energy consumption between 2000 and 2021 is 3.413% (Russian Federation), 10.186% (Mexico), 14.014% (Türkiye), 16.345% (China), 33.727% (Indonesia), 38.505% (India), and 45.359% (Brazil) (67). In conclusion, the findings demonstrate that the stringency of environmental policy and the use of renewable energy play a key role in the effect of CO_2_ emissions on LE60 and HALE60 in the E7 countries.

Income is one of the fundamental factors in achieving a longer lifespan by maintaining a healthy life and accessing higher-quality medical care. This theoretical expectation has been largely confirmed by nearly all studies, including Anwar et al. (14), Aytemiz et al. (17), Magazzino et al. (18), Chen et al. (22), Radmehr and Adebayo (25), Țarcă et al. (29), Miladinov (42), Şenol et al. (43), Shi et al. (44), Morina et al. (45), and Karunarathne et al. (46) to a great extent. Similarly, our results also demonstrate that per capita GDP is a significant positive determinant of both LE60 and HALE60. China, India, Türkiye, Indonesia, and Russia have achieved significant increases in per capita GDP between 2000 and 2021, but the effect of per capita GDP on LE60 and HALE60 is largest in the Russian Federation, Mexico, Brazil, India, and China. Therefore, Türkiye and Indonesia experienced a relatively lower positive effect of increases in GDP on LE60 and HALE60 when compared with other E7 countries.

Last, health expenditures are anticipated to positively influence life expectancy by means of better health facilities and physicians, and the results of Anwar et al. (14), Bayar et al. (16), Aytemiz et al. (17), Radmehr and Adebayo (25), Morina et al. (45), Rezapour et al. (48), Bunyaminu et al. (49), Sarıyıldız (50), and Alimi et al. (51) also support these theoretical views. Our results demonstrate that both general government and private health expenditures positively impact LE60 and HALE60 in Brazil, China, India, the Russian Federation, and Türkiye. The positive effect of public and private health expenditures on LE60 and HALE60 is relatively larger in Türkiye and the Russian Federation than in India, China, and Brazil, and is insignificant in Indonesia and Mexico. The average share of public health expenditures in total health expenditures in Türkiye and the Russian Federation is 74.5 and 61.4%, respectively, between 2000 and 2021. However, this ratio is 48.0% in Mexico, 45.6% in China, 43.0% in Brazil, 37.2% in Indonesia, and 25.7% in India. Therefore, Türkiye and the Russian Federation, with their public sector-dominated healthcare systems, perform better than countries with equal shares of public and private healthcare systems. Furthermore, the level of current health expenditures in the E7 countries is much lower than that in developed countries. In conclusion, these differences between countries can result from the inefficiency of health and social security systems, inequalities in income distribution, and relatively low health expenditure.

Theoretically, a mutual interaction between LE60, HALE60, CO_2_ emissions, per capita GDP, and health expenditure is anticipated. However, few studies have performed two-way analyses of these variables. In this regard, Rahman et al. (23), Javanshirova (26), Khan et al. (34), Rjoub et al. (35), and Hasnawati et al. (41) conducted a two-way analysis between LEB and CO_2_ emissions. Rahman et al. (23), Javanshirova (26), and Rjoub et al. (35) determined a unilateral causal connection from CO_2_ emissions to LEB, and Khan et al. (34) disclosed a bidirectional causal connection between LEB and carbon dioxide emissions, while Hasnawati et al. (41) unveiled an insignificant interplay between the two variables. In contrast, Magazzino et al. (18) and Alwago (47) performed a two-way analysis between LEB and per capita GDP. Magazzino et al. (18) unveiled a unidirectional causality from LEB, LE40, and LE60 to per capita GDP, and Alwago (47) disclosed an insignificant causal link between LEB and per capita GDP. Finally, Bayar et al. (16) conducted a two-way analysis between health expenditures and LEB and discovered unidirectional causality from LEB to health expenditures. The outcomes of our causality analysis indicate that LE60 and HALE60 also have a significant effect on CO_2_ emissions, per capita GDP, and health expenditure, unlike the results of these limited studies.

## 6 Conclusions, limitations, policy implications, and future research directions

The share of older adults in the global population has been increasing steadily, and this changing population structure has important economic, social, environmental, and health implications for societies. Nevertheless, most studies have focused on the effects of various economic, social, and environmental factors on life expectancy at birth (LEB). To address this gap, the present study investigates the effects of CO₂ emissions, per capita GDP, and public and private health expenditures on LE60 and HALE60 by employing cointegration methods with structural breaks and causality tests.

The study period covers the period 2001–2021 due to the data availability of LE60 and HALE60. Furthermore, the E7 countries constitute our sample, and the results of the study are widely specific to these countries.

The results of the causality analysis indicate a bidirectional relationship between LE60, HALE60, CO₂ emissions, public and private health expenditures, and GDP, which is consistent with theoretical expectations. Furthermore, the long-term analysis demonstrates that CO₂ emissions negatively affect LE60 and HALE60, whereas per capita GDP and public and private health expenditures positively influence both.

Based on our empirical findings, several policy implications emerge. First, the stringency of environmental policies is very low in Mexico, Brazil, Russia, and Indonesia. Furthermore, the share of renewable energy use in the total energy consumption is very low in the Russian Federation, Mexico, Türkiye, and China. Therefore, these countries should increase the stringency of their environmental policies through legal and market-based instruments such as environmental taxes and tradable permits. Furthermore, the development of renewable energy technologies should be prioritized in these countries. Increases in per capita GDP led to improvements in LE60 and HALE60 in all E7 countries, but Türkiye and Indonesia especially experienced a relatively lower positive effect of increases in per capita GDP on LE60 and HALE60 compared with the other E7 countries. Therefore, these countries should improve income inequality through redistributive policies such as transfer payments and a progressive tax system. Finally, most E7 countries experience a positive effect of public and private health expenditures on LE60 and HALE60, but the health gains of Türkiye and the Russian Federation are relatively larger than those of India, China, and Brazil. Therefore, institutional arrangements to improve the efficiency of health and social security systems, redistributive policies, and further investments in health infrastructure are crucial for increasing the health benefits of public and private health expenditures. Finally, the results of this study imply that the stringency of environmental policies, use of renewable energy, efficiency of health and social security systems, and income redistribution are significant factors in the interaction between CO_2_ emissions, per capita GDP, public and private health expenditures, and older adults’ health. Therefore, future research should focus on examining the mediating impact of these factors on the nexus between older adults’ health, per capita GDP, and public and private health expenditures.

## Data Availability

The raw data supporting the conclusions of this article will be made available by the authors, without undue reservation.
